# Exploring EEG-based motor imagery decoding: a dual approach using spatial features and spectro-spatial Deep Learning model IFNet

**DOI:** 10.3389/fninf.2024.1345425

**Published:** 2024-02-29

**Authors:** Javier V. Juan, Rubén Martínez, Eduardo Iáñez, Mario Ortiz, Jesús Tornero, José M. Azorín

**Affiliations:** ^1^Brain-Machine Interface Systems Lab, Universidad Miguel Hernández de Elche, Elche, Spain; ^2^Center for Clinical Neuroscience HLM, Hospital Los Madroños, Brunete, Spain; ^3^Universidad Autónoma de Madrid, Madrid, Spain; ^4^INNTEGRA, Hospital Los Madroños, Brunete, Spain; ^5^Instituto de Investigación en Ingeniería de Elche-I3E, Universidad Miguel Hernández de Elche, Elche, Spain; ^6^ValGRAI: Valencian Graduated School and Research Network of Artificial Intelligence, Valencia, Spain

**Keywords:** brain-machine interface (BMI), electroencephalography (EEG), motor imagery (MI), deep learning (DL), convolutional neural network (CNN), common spatial patterns filter bank (CSPFB), linear discriminant analysis (LDA), IFNet

## Abstract

**Introduction:**

In recent years, the decoding of motor imagery (MI) from electroencephalography (EEG) signals has become a focus of research for brain-machine interfaces (BMIs) and neurorehabilitation. However, EEG signals present challenges due to their non-stationarity and the substantial presence of noise commonly found in recordings, making it difficult to design highly effective decoding algorithms. These algorithms are vital for controlling devices in neurorehabilitation tasks, as they activate the patient's motor cortex and contribute to their recovery.

**Methods:**

This study proposes a novel approach for decoding MI during pedalling tasks using EEG signals. A widespread approach is based on feature extraction using Common Spatial Patterns (CSP) followed by a linear discriminant analysis (LDA) as a classifier. The first approach covered in this work aims to investigate the efficacy of a task-discriminative feature extraction method based on CSP filter and LDA classifier. Additionally, the second alternative hypothesis explores the potential of a spectro-spatial Convolutional Neural Network (CNN) to further enhance the performance of the first approach. The proposed CNN architecture combines a preprocessing pipeline based on filter banks in the frequency domain with a convolutional neural network for spectro-temporal and spectro-spatial feature extraction.

**Results and discussion:**

To evaluate the approaches and their advantages and disadvantages, EEG data has been recorded from several able-bodied users while pedalling in a cycle ergometer in order to train motor imagery decoding models. The results show levels of accuracy up to 80% in some cases. The CNN approach shows greater accuracy despite higher instability.

## 1 Introduction

Brain-machine interfaces (BMIs) enable the recording of neural activity from the user's brain and its utilization as a control element for devices (Lebedev and Nicolelis, 2006; Lebedev, 2014; Slutzky, 2019). These interfaces can employ invasive methods (which involve recording the signals directly from the brain) such as electrocorticography (ECoG) (Leuthardt et al., 2006a,b), or invasive electroencephalography (iEEG) (Holdgraf et al., 2019; Balaji and Parhi, 2022); or non-invasive methods (record the signals through the scalp) like electroencephalographic signals (EEG) (Sanei and Chambers, 2007; Garipelli et al., 2013; Ortiz et al., 2020), functional Magnetic Resonance Imaging (fMRI) (Heeger and Ress, 2002; Misaki et al., 2021), and magnetoencephalography (MEG) (Supek and Anie, 2014; Hillebrand et al., 2019).

In the field of motor rehabilitation, Brain-Machine Interfaces play a pivotal role in detecting the patient's intention to move (Mak and Wolpaw, 2009). This facilitates the control of clinical devices, such as lower limb exoskeletons (Contreras-Vidal et al., 2016) or cycle ergometers (Comani et al., 2013; Ortiz et al., 2019b). While the former provides an experience closer to natural walking, the latter offers advantages in terms of safety, ease of use, and cost-effectiveness. Motor imagery (MI) is a commonly employed technique in these BMIs to activate the brain's motor area and improve rehabilitation outcomes. This is primarily attributed to two main reasons. First, certain studies suggest that the human motor cortex is particularly active during walking (Castermans and Duvinage, 2013). Second, deeper patient engagement in their tasks can lead to increased activation of the affected motor area, benefiting not only their muscle therapy through actual limb movement (supported by a cycle ergometer, exoskeleton, etc.) but also their neural, spinal and nervous rehabilitation. This heightened mental involvement is fostered through MI-based control. In this regard, EEG-based Brain-Machine Interfaces are particularly advantageous, primarily because of the ease of user instrumentation and the minimal constraints on patients. Certainly, numerous studies have delved into motor imagery decodification in recent decades (Ang and Guan, 2008). For example, Xu et al. utilized discrete wavelet transform to classify EEG signals during left-hand and right-hand motor imagery (Xu and Song, 2008). Another illustration is provided by Shen et al. in their exploration of motor imagery-EEG-based gait rehabilitation control (Shen et al., 2022). Notably, there are studies dedicated to pedaling motor imagery decodification from EEG signals, such as the work by Ortiz et al. (2019b) and Delisle-Rodriguez et al. (2019), among others. These investigations are unified in their objective of decoding motor imagery from EEG signals, employing a diverse array of techniques and algorithms, and achieving promising results in certain instances. However, EEG signals are characterized by a substantial signal-to-noise ratio, which complicates their analysis (Kumar and Bhuvaneswari, 2012).

Approaches for decoding MI from EEG signals typically involve extracting discriminative features, whether they are temporal, spatial, or spectral in nature, and feeding them to a classifier (Lotte et al., 2018). Additionally, Convolutional Neural Networks (CNNs) based on Deep Learning (DL) have been explored as potential solutions. However, further evidence is still needed to establish whether they outperform feature-based methods (Lawhern et al., 2018). In the specific case of decoding motor imagery from EEG, spatial features based on the Common Spatial Patterns (CSP) algorithm have proven to yield good results (Ortiz et al., 2020). Additionally, frequency band division has shown to be effective in enhancing MI decoding models (Ortiz et al., 2020). Furthermore, in the case of Ortiz et al. (2020), it appears that these features are particularly well-suited for Bayesian linear classification algorithms like Linear Discriminant Analysis (LDA). There are also some neural networks optimized for MI decoding from EEG signals, such as Autthasan et al. (2021), based on spatial features, and Wang et al. (2023), which combines the concept of a filter bank with spatial feature analysis.

For all these reasons, this study has compared the performance of the method based on spatial feature classification developed in Juan et al. (2022), with an alternative approach grounded in DL, embodied within the IFNet neural network framework (Wang et al., 2023). The optimal solution for motor imagery decoding, whether achieved through traditional feature extraction methods or DL approaches, has the potential to enhance the quality of life for millions of individuals with motor disabilities around the globe.

## 2 Materials and methods

### 2.1 Users

For this case study, data from thirteen able-bodied users were employed. Six female users (U01, U03, U06, U08, U09 and U11) and seven male users (U02, U04, U05, U07, U10, U12 and U13), all without any diagnosed motor or neurological dysfunction, within selection criteria, with ages ranging from 18 to 62 years (32±13). All users were provided with clear information about the study and signed the informed consent, which was approved by the Comité de Ética de la Investigación con medicamentos of the Hospital Universitario Severo Ochoa from Leganés (Comunidad de Madrid, Spain) under the code HLM-CYCLING-EEG.

### 2.2 Experimental protocol

The protocol was strategically designed to train two types of control: rest control (with the cycle ergometer inactive) and motion control (with the cycle ergometer active). This involved having relaxation phases (without Motor Imagery, MI) and MI phases, both at rest and during pedaling (with the device assisting the user as in real therapy).

**Figure 1:**
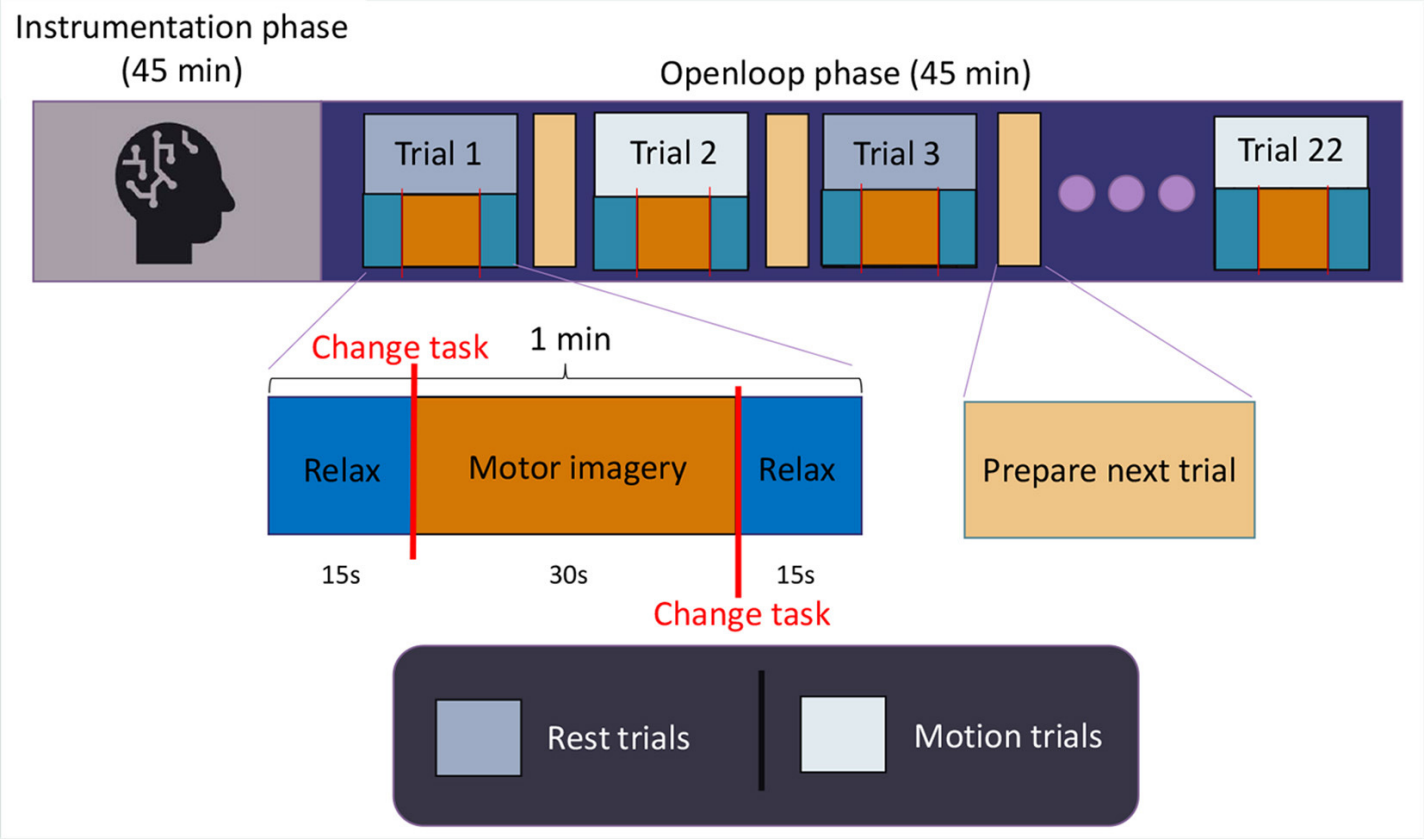
Experimental protocol. Each user completed four sessions of this protocol.

Each test session consisted of 22 trials, with each trial comprising 15 seconds of relaxation, followed by 30 seconds of MI during pedaling, and then another 15 seconds of relaxation. Users were acoustically alerted at each task transition, resulting in two-second intervals introduced between tasks to exclude those data segments and prevent interference from external stimuli. Out of the 22 trials, the odd-numbered ones were conducted with the cycle ergometer inactive, and the even-numbered ones with the cycle ergometer active in passive mode. This provided data on the initiation commands (MI at rest) and cessation commands (relaxation during motion) for the rehabilitation device in a potential therapy setting. Every user conducted four of these sessions in four consecutive days, one session per day. Figure 1 illustrates a schematic of the employed protocol.

A total of 22 trials per session were selected, with the goal of having 11 trials from each category (rest and motion), allowing for an 11-fold cross-validation (10+1) for each paradigm in every test session, and generating CSP-LDA models. Consequently, the lowest-performing trial from each paradigm in each session can be excluded, and the remaining 10 resting trials can be used for training IFNet models. This procedure will be further explained in Section 2.4.1.3. Each user participated in four test sessions, with the intention of acquiring an adequate volume of data for training the convolutional models without overly burdening the users, thus preventing a decrease in their performance. Prior to each user's first session, they were provided with guidelines on how to perform motor imagery, with an emphasis on guiding them toward a kinesthetic and consistent form of imagination across the test sessions and to remain quiet during the registers. Additionally, they completed the Movement Imagery Questionnaire-3 in its Spanish version (Trapero-Asenjo et al., 2021), as all users were Spanish speakers. Table 1 depicts the results obtained by each user. The users were also provided with basic guidelines for achieving a correct state of relax, with an emphasis on the importance of contrasting it with the motor imagery state.

**Table 1:** Motor imagery indexes obtained by every user after filling the MIQ-3 questionnaire in its Spanish version (Trapero-Asenjo et al., 2021).

**User**	**External visual MI index**	**Internal visual MI index**	**Kinesthetic MI index**	**User**	**External visual MI index**	**Internal visual MI index**	**Kinesthetic MI index**
U01	6.00	5.50	7.00	U08	6.50	6.75	3.00
U02	5.75	6.25	4.75	U09	5.25	5.50	6.75
U03	5.75	6.00	6.00	U10	5.25	7.00	6.50
U04	5.50	1.50	2.00	U11	5.75	6.75	5.50
U05	4.00	5.00	3.25	U12	2.75	3.25	2.75
U06	5.00	5.50	6.75	U13	7.00	7.00	6.75
U07	5.25	5.00	5.25				

### 2.3 Experimental setup

The EEG recording equipment used in the experimental sessions of this project consists of a 32-channel g.NAUTILUSPROFlexible cap with the g.SCARABEO electrode distribution, along with the Wi-Fi HEADSET transmitter and BASE STATION receiver, all from the manufacturer g.tec medical Engineering GmbH. The reference electrode was clipped to the right earlobe, which was previously cleaned with skin prep gel, and all electrodes were accompanied by conducting gel to achieve an appropriate impedance level for recording, ensuring a clean signal quality. The signals were recorded at 500Hz.

Regarding the cycle ergometer, the model used is the CycleMotus™ A4, developed by Fourier Intelligence ©. The screen was covered at all times to prevent external stimuli. The rotation speed was adjusted according to each user's preference, as we aim to develop adaptable and robust models that can meet the specific needs of each patient for potential future implementation in real therapy. In any case, all users selected rotation speeds within the range of 20 to 40 revolutions per minute and maintained these speeds throughout all four test sessions.

**Figure 2:**
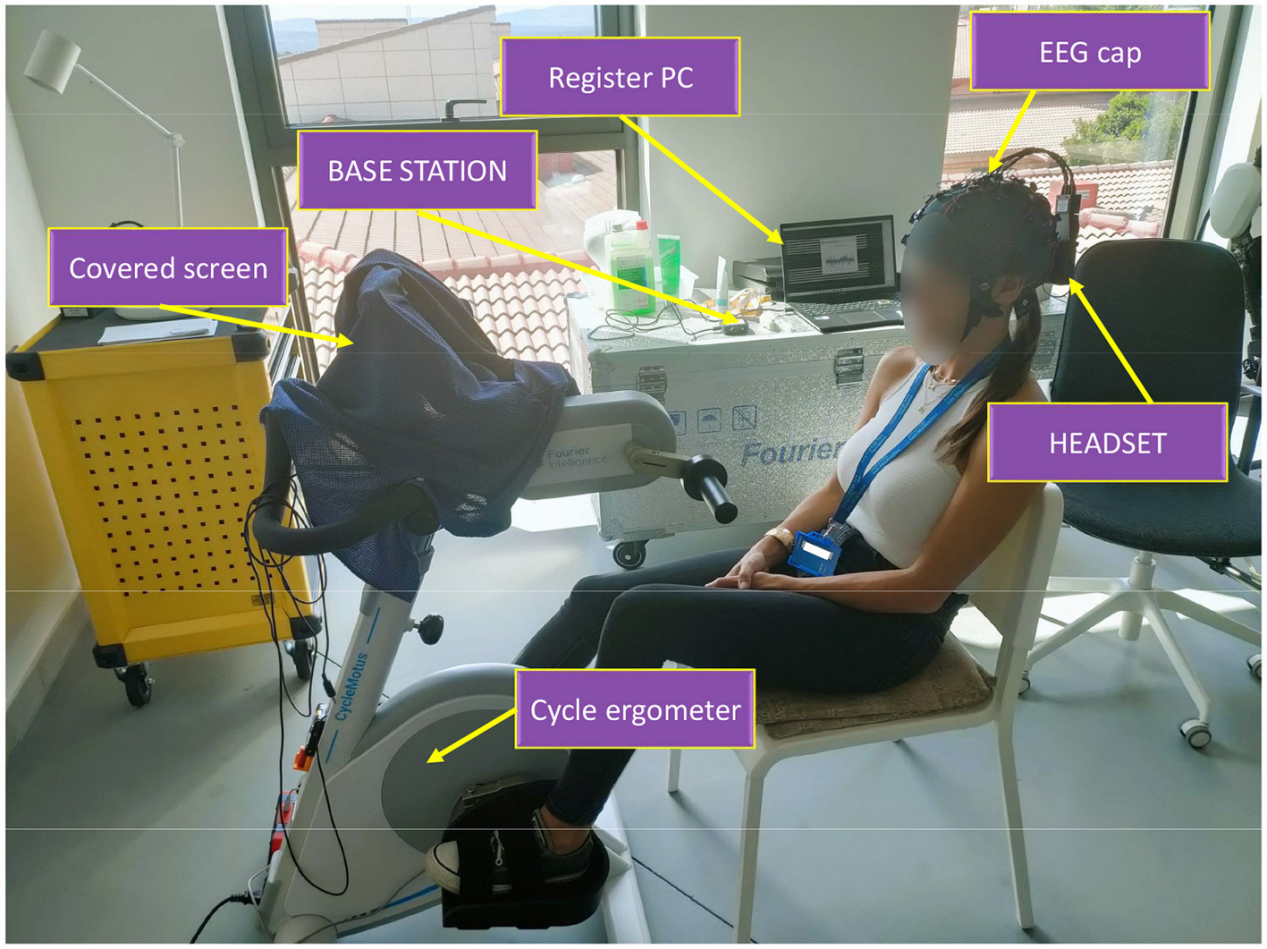
Experimental setup employed in the tests.

Figure 2 shows the experimental setup.

### 2.4 Motor imagery decodification methods

In this section, we describe the two selected methods for motor imagery decoding. Method I involves feature extraction using Common Spatial Patterns followed by classification using Linear Discriminant Analysis, while Method II is based on a Convolutional Neural Network algorithm, IFNet.

#### 2.4.1 Method I: CSP-LDA

##### 2.4.1.1 Preprocessing

**Figure 3:**
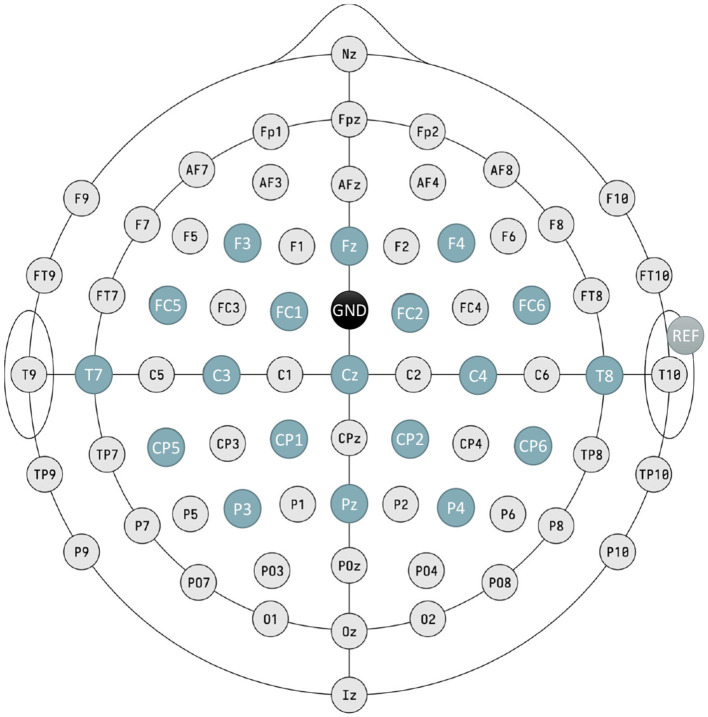
Schematic of electrode placement; blue electrodes form the ones used in the study. The ground electrode (GND) is situated at FCz, whereas reference electrode (REF) is clipped to the right earlobe.

For this first analysis method, a series of hardware filters were applied to all channels during signal acquisition, as implemented by the manufacturer. This includes a noise reduction algorithm integrated into the device, a 4th-order Notch filter between 48 Hz and 52 Hz to eliminate the 50 Hz power line component, and an 8th-order band-pass filter with cut-off frequencies of 0.5 Hz and 100 Hz to confine the signal to the desired frequency range. Following this, a preselection of channels from the motor area was performed, which comprises 19 electrodes. According to the 10/10 EEG standard, these electrodes are: FZ, F4, F3, FC5, FC1, FC2, FC6, T7, C3, CZ, C4, T8, CP5, CP1, CP2, CP6, P3, PZ, and P4 (Figure 3).

**Figure 4:**
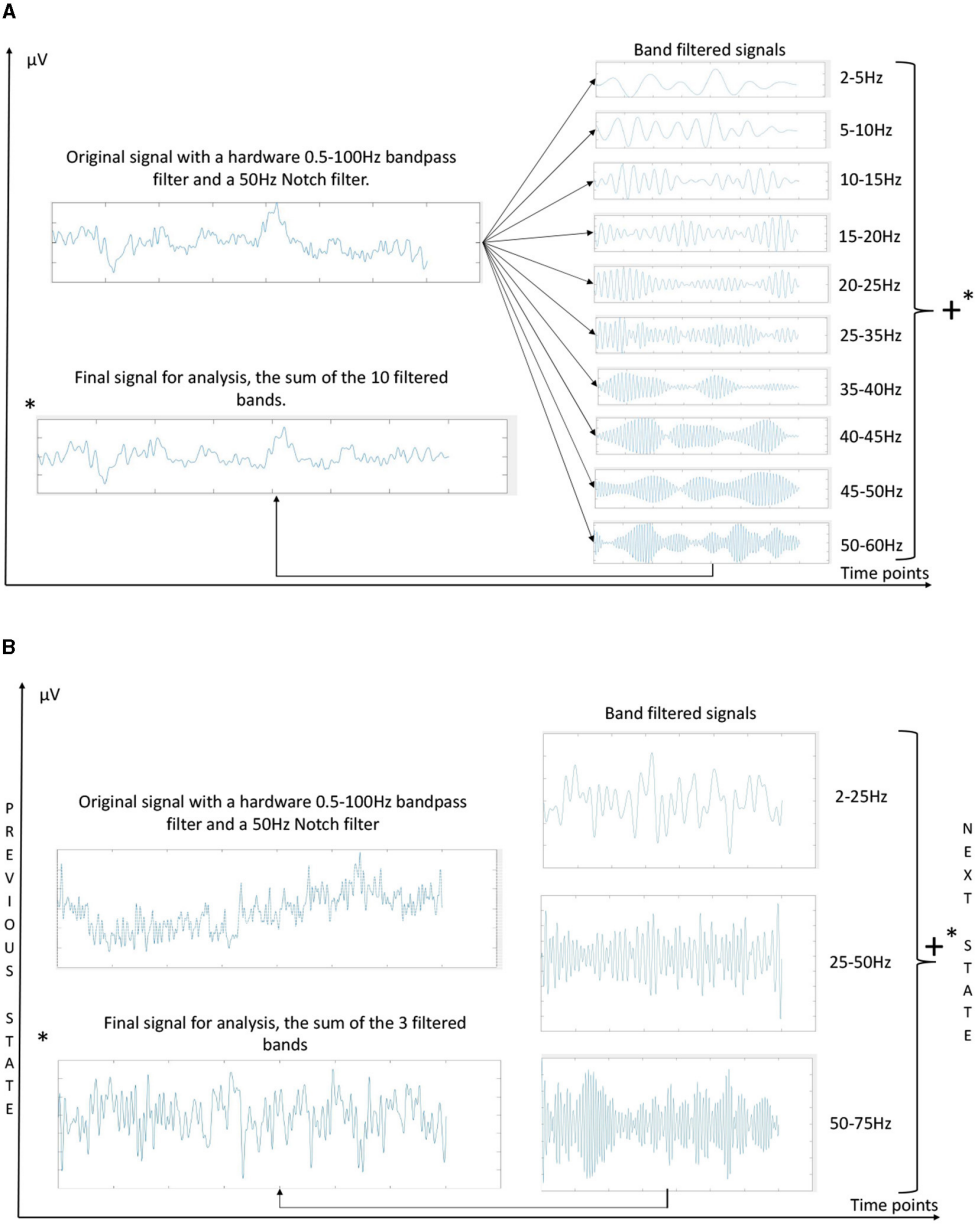
Preprocessing techniques employed in the analysis, **(A)** for Method I, and **(B)** for Method II. The primary distinctions lie in the number of frequency bands and the filtering approach. In **(A)** the entire signal is filtered simultaneously, while in **(B)** one-second time windows are filtered, with the filter state updated for the subsequent temporal window. **(A)** Filter bank schematic used for MI decoding method I (CSP+LDA). **(B)** Filter bank schematic used for MI decoding method II (IFNet). ^*^indicate that the graph marked with it is the sum of the graphs on the right of the figure.

Next, a frequency band division was applied for filtering, consisting of 10 distinct bands: 2–5Hz, 5–10Hz, 10–15Hz, 15-20Hz, 20–25Hz, 25–35Hz, 35–40Hz, 40–45Hz, 45–50Hz, 50–60Hz. For each of the selected channels, the signal was simultaneously filtered within each of these ranges using second-order Butterworth filters. The resulting signals from each channel were summed to obtain the signal for analysis, following the method employed in Wang et al. (2023), as illustrated in Figure 4.

##### 2.4.1.2 Feature extraction

**Figure 5:**
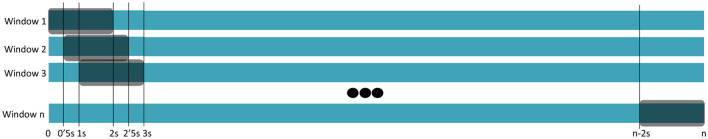
Temporal window segmentation utilized in the analysis. Each window is labeled (MI or relax).

This part of the analysis is based on the CSP filter. This filter was applied to each of the 19 preprocessed selected channels, preceded by a temporal windowing process. The windows have a duration of two seconds with an overlap of one and a half seconds, resulting in feature extraction every half second, for each of the 19 EEG channels. The label assigned to each window is determined by its mode, with windows showing a mode indicative of a task change being excluded from the analysis. Figure 5 provides a more visual representation of this temporal segmentation.

##### 2.4.1.3 Classification

**Figure 6:**
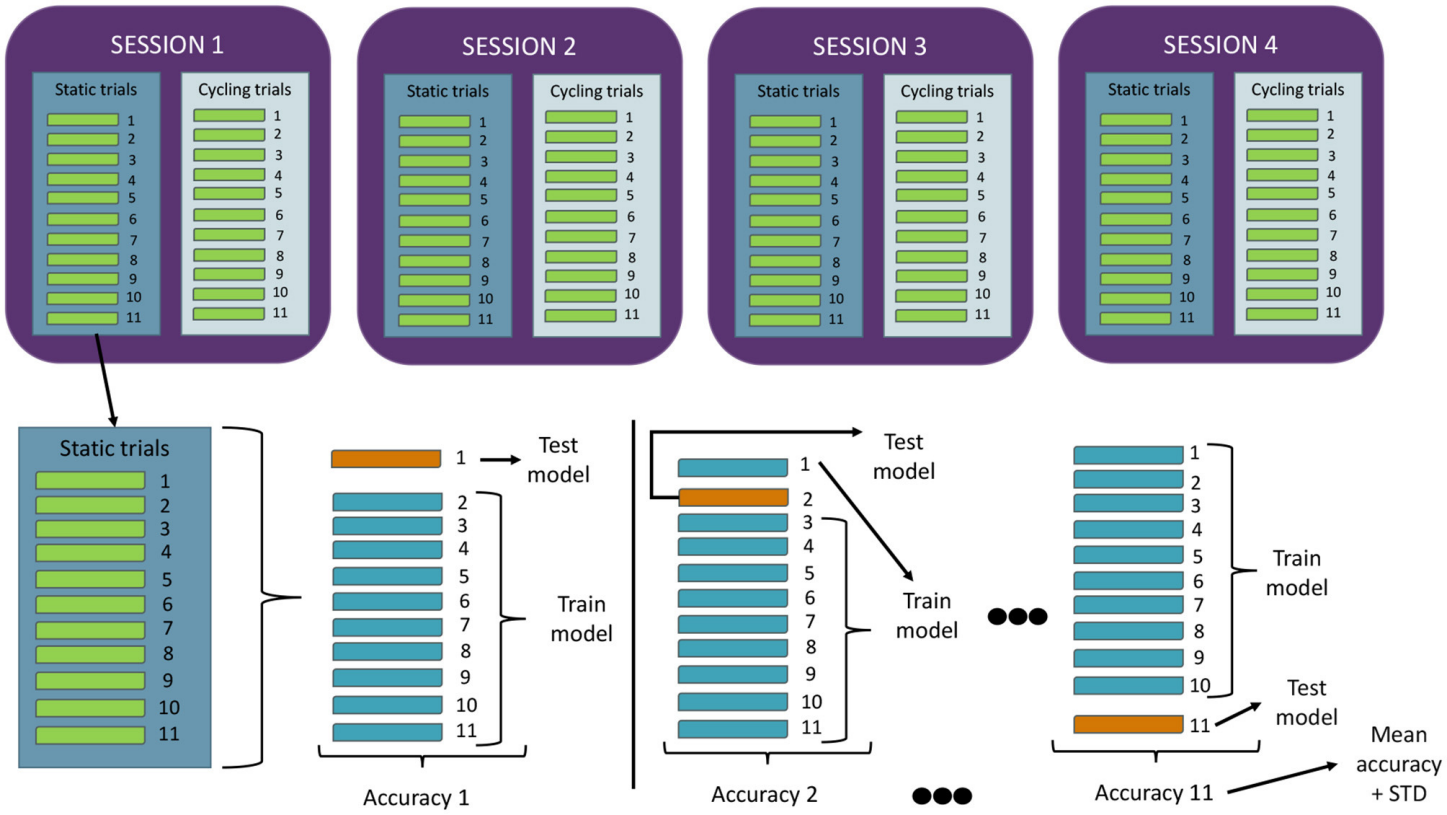
Cross-fold validation implemented for the analysis. The figure illustrates the data distribution schematic for a single user and how the various motor imagery decoding models are generated. For each of these models (two per session, one for static and one for cycling), a 10+1 cross-validation was employed. This involved using each trial as a test case and collecting standard deviation results in this manner.

The temporal window classification was performed using the LDA algorithm, feeding it with the features obtained in the previous step, from all 19 channels together. The algorithm provides an output (MI or relax) every half second, which can be compared with the actual label to check for accuracy. Figure 6 illustrates the 10+1 cross-validation model applied during the data analysis with CSP-LDA method. This cross-fold validation model has been implemented in every of the eight decodification models presented by every user (static and cycling, for each one of the four experimental sessions conducted by every user). Every model is created with ten trials and tested with the eleventh one, and this is repeated eleven times so that every trial has been employed as the test one. Hence, standard deviation data can be computed with the accuracy results got from every model.

In the context of our binary classifier, we opted for accuracy as a performance metric due to the balanced nature of the datasets. The choice of accuracy provides a straightforward measure of the classifier's correctness in distinguishing between the two classes. Precision focuses on the accuracy of the positive predictions, recall assesses the ability of the classifier to capture all positive instances, whereas other metrics such as F1-score balances precision and recall. Additionally, the implementation of cross-validation serves the purpose of obtaining standard deviation data, contributing to a more comprehensive evaluation of the classifier's consistency and robustness across different subsets of the dataset. This metric selection aligns with the balanced distribution of classes and ensures a holistic assessment of the classifier's performance.

#### 2.4.2 Method II: IFNet

##### 2.4.2.1 Preprocessing

The signal acquisition process was conducted in the same manner as in Method I (Section 2.4.1.1), utilizing the same hardware filters and channel preselection (Figure 3); it involves the same records. Regarding the filter bank, a different approach was employed for this model, focusing more on real-time analysis. In this case, it consisted of an online triple Butterworth filter of the 8th order in the state space, filtering one-second windows in series, in parallel across these three frequency bands: 2–25Hz, 25–50Hz, and 50–75Hz. After filtering each window, the results from the three branches are summed to obtain the final signal for analysis (Figure 4), emulating the preprocessing technique from Wang et al. (2023). This filtering strategy is tailored for real-time processing, mirroring a real therapy scenario. The selection of an state space filter, which is better suited for processing acquired temporal windows rather than the entire recorded signal, and the reduction of the number of frequency bands to three, were driven by computational efficiency and the demands of real-time applications.

After filtering the signals, we applied the MinMax scaler to standardize them for preparing the datasets for the neural network. It not only improved the data's consistency but also set the stage for potential future enhancements. These enhancements could include adding synthetic samples, calibrating the system with conformal prediction, or fine tuning algorithms.

##### 2.4.2.2 Classification

**Figure 7:**
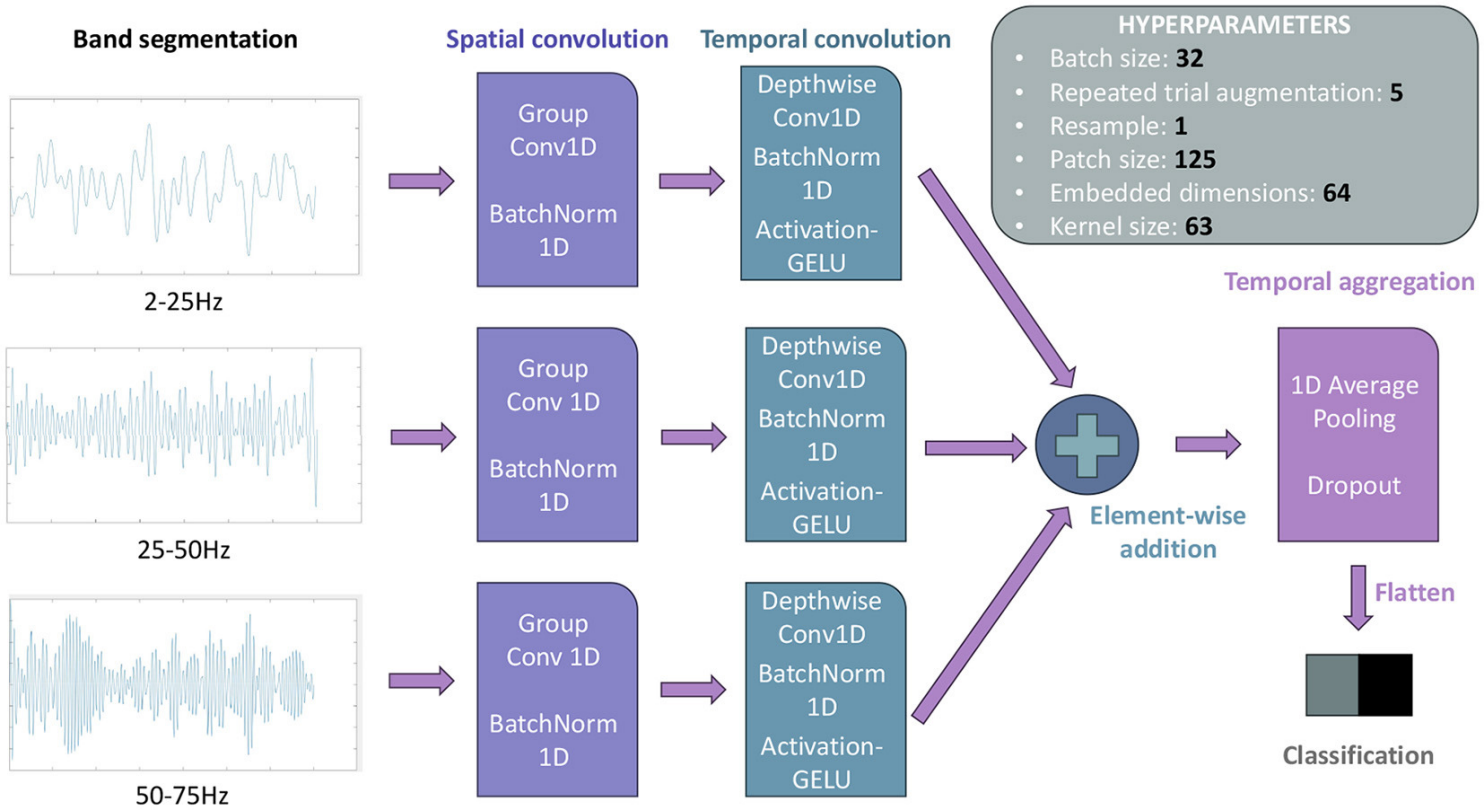
Overview of the configuration settings applied to the IFNet neural network in this study. For every model, 1000 epochs were computed followed by a 500 epochs retraining, focusing on reducing the validation loss. More information about its structure and functionalities can be found in Wang et al. (2023).

In this method, the same window division was applied as in Method I (two-second windows with a one and a half-second overlap, resulting in a classifier output every half second, Figure 5). Using this windowing approach, the IFNet network (Wang et al., 2023) was trained, creating a model for each user by dividing all their preprocessed data into 80% for training, 15% for validation, and 5% for testing. In terms of functionality and hyperparameters, no changes were made to the default network, except for adjusting the filters to match those used in preprocessing; Figure 7 provides a brief summary of this configuration and IFNet's body, which involves an spatial convolution followed by a temporal one, preparing the data for classification. The worst trial from each session of recordings, as selected by Method I (see Section 2.4.1.3), was removed, leaving 10 trials for each of both the static and cycling models.

Again, as datasets are balanced, accuracy has been chosen as evaluation metric. It is important to note that by using this approach, each user has only two models: one for the static condition and one for cycling. To create these models, data from all four experimental sessions were used for training, resulting in inter-session models formed by 40 static trials and by 40 cycling trials. Convolutional Neural Networks tend to improve their performance as the amount of data increases (provided overfitting is avoided), which justifies this inter-session recording and training. This configuration leads to approximately 1,825 EEG windows for training each model, 350 for validation, and 115 for testing, for every user. However, it's noteworthy to mention that the training times for this approach are considerably longer compared to Method I. Training each model in Google Colab takes approximately 40 minutes, while the training process in Method I is nearly instantaneous on a laptop (ACER Aspire 5, Intel CORE i7 10th gen, 8GB RAM) using MATLAB. Consequently, cross-validation is not implemented in this case.

### 2.5 Frequency bands ablation study

To evaluate the impact of different signal filtering frequency bands on motor imagery decoding in Method II (IFNet), an ablation study was conducted. This study was conducted with three different users: U01, who exhibited high accuracy with Method I (CSP-LDA) but a significant difference between MI and relax accuracy when using IFNet; U07, who showed outstanding results with both decoding approaches; and U10, who achieved average results. For each of these users, four new datasets were created, trained, and tested using IFNet, following the MI decoding method II. The only variation was the adjustment of the frequency bands in the state space filter (see Section 2.4.2.1). This allowed us to assess the effects of different frequency bands on motor imagery decoding accuracy.

In the first case, only the lowest frequency band (02–25 Hz) was applied but divided into two consecutive bands: 02–14 and 14–25Hz. This division allowed us to assess the impact of the lowest band on the relax task decoding accuracy and whether reducing the number of frequency bands could help mitigate the class accuracy imbalance. Secondly, the highest band (high gamma, 50–75 Hz) was removed to measure its contribution to the analysis. The middle band (high beta, low gamma, 25–50 Hz) was eliminated in the next scenario to quantify its role in motor imagery class decoding and to examine how using two non-consecutive frequency bands for filtering affects decoding, particularly in terms of class accuracy bias. Finally, the lowest band (2–25Hz) was also removed. This ablation study provided preliminary insights into the contributions of each frequency band to the task decoding and its potential for optimization, as discussed in Section 4.2.

## 3 Results

### 3.1 IFNet and CSP-LDA comparison

**Figure 8:**
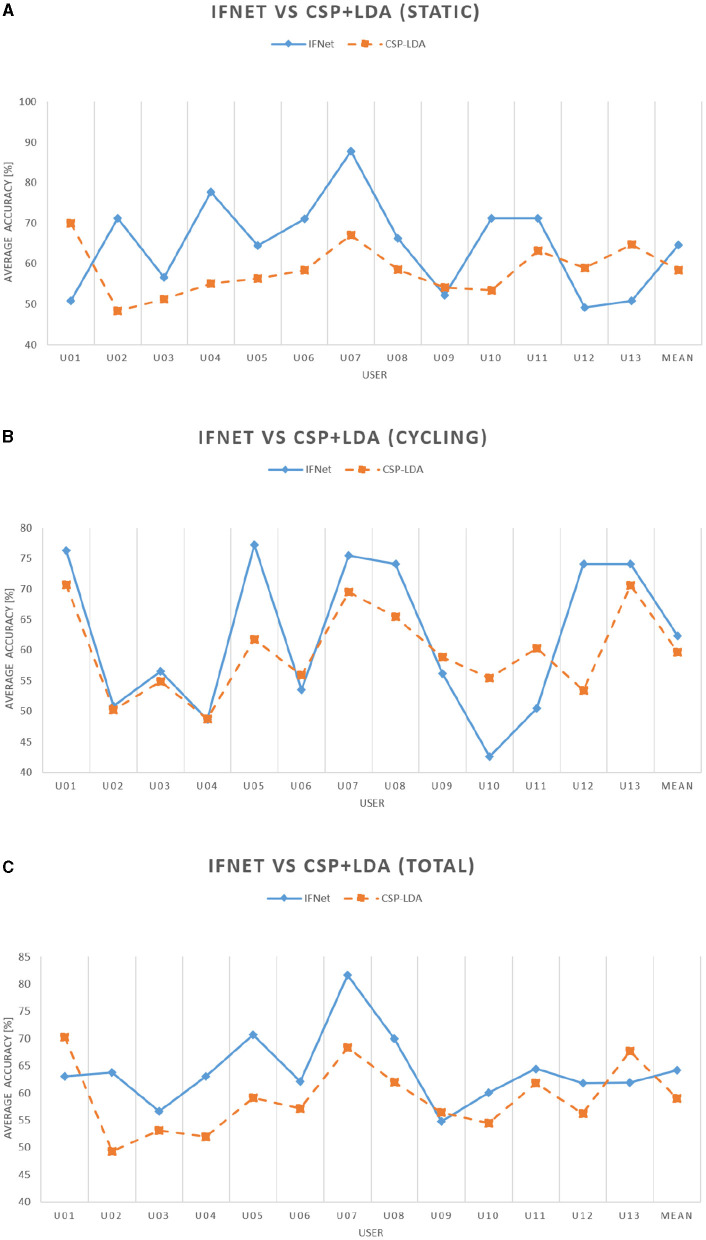
Inter-user performance comparison between the IFNet and CSP-LDA classifiers. **(A)** Inter-user static performance comparison between the IFNet and CSP-LDA classifiers (correlation coefficient of -0.06). **(B)** Inter-user cycling performance comparison between the IFNet and CSP-LDA classifiers (correlation coefficient of 0.72). **(C)** Inter-user average performance comparison between IFNet and CSP-LDA classifiers (correlation coefficient of 0.42).

In this section, we present the outcomes of a series of offline tests conducted using CSP-LDA and IFNet classifiers. Table 2 provides a comparative analysis of these decoding methods following four postulations: static relax, static MI, cycling relax and cycling MI, revealing a noteworthy accuracy enhancement achieved with IFNet over the conventional state-of-the-art approach. As a cross-fold validation has been applied using the CSP-LDA classification method, the results are also presented with standard deviation data. The average classification accuracy with IFNet surpasses the traditional method by nearly 5%, with accuracy improvements observed in 10 out of the 13 users. This feature is even more pronounced when comparing static data, as the improvement reaches the 6%, in decrease of the approximately 3% achieved with cycling models. These aspects are further illustrated in Figure 8, which showcases a trend between both curves. It is important to note, however, that when employing IFNet, there is a notable increase in the variability of accuracy between the two classes for each user, presenting alarmingly high average standard deviation values. A substantial variability in decoding performance among users is a common observation in EEG processing. In this context, it appears that CSP-LDA exhibits a marginally greater degree of stability. There don't appear to be significant performance discrepancies between both classifiers in the context of the four postulates, except for the noteworthy improvement in cycling relax accuracy achieved with CSP-LDA. The results for the other three scenarios reinforce the overall superiority of the IFNet classifier in terms of average accuracy, as previously discussed, particularly in the case of cycling motor imagery, showcasing an accuracy improvement of over 11%.

**Table 2:** Results of the classification presented in Section 2.

**USER**	**Task**	**IFNet (% accuracy)**	**CSP-LDA (% accuracy±STD)**	**USER**	**Task**	**IFNet (% accuracy)**	**CSP-LDA (% accuracy±STD)**
U01	Static relax	0.0	68.5 ± 7.2	U08	Static relax	54.5	57.3 ± 14.2
	Static MI	100.0	71.3 ± 9.9		Static MI	77.6	59.8 ± 13.3
	Cycling relax	66.1	69.4 ± 8.0		Cycling relax	58.0	71.2 ± 14.8
	Cycling MI	86.2	77.0 ± 9.9		Cycling MI	89.7	59.6 ± 16.1
	Mean	63.1	70.3 ± 12.4		Mean	69.9	62.0 ± 13.6
U02	Static relax	93.8	43.2 ± 13.2	U09	Static relax	89.3	56.1 ± 7.2
	Static MI	16.4	50.6 ± 11.8		Static MI	77.6	52.1 ± 11.9
	Cycling relax	97.3	56.9 ± 20.2		Cycling relax	54.5	66.0 ± 17.5
	Cycling MI	16.4	71.3 ± 9.9		Cycling MI	77.6	51.7 ± 9.5
	Mean	63.8	49.3 ± 8.2		Mean	54.8	56.5 ± 10.3
U03	Static relax	57.1	52.9 ± 8.2	U10	Static relax	93.8	56.9 ± 7.9
	Static MI	56.0	49.7 ± 9.5		Static MI	61.5	50.0 ± 10.7
	Cycling relax	67.0	58.4 ± 9.0		Cycling relax	44.6	59.5 ± 12.0
	Cycling MI	46.6	51.5 ± 12.1		Cycling MI	40.5	51.4 ± 10.7
	Mean	56.7	53.1 ± 8.7		Mean	60.1	54.5 ± 11.0
U04	Static relax	71.4	57.1 ± 10.2	U11	Static relax	93.8	63.5 ± 8.5
	Static MI	83.6	53.3 ± 12.0		Static MI	61.5	63.0 ± 11.1
	Cycling relax	42.9	52.6 ± 11.8		Cycling relax	99.1	68.0 ± 9.7
	Cycling MI	54.3	45.0 ± 10.0		Cycling MI	3.5	52.7 ± 13.7
	Mean	63.1	52.0 ± 10.4		Mean	64.5	61.8 ± 9.6
U05	Static relax	45.5	60.1 ± 13.9	U12	Static relax	89.3	63.0 ± 11.4
	Static MI	82.8	52.6 ± 14.1		Static MI	10.3	55.1 ± 13.9
	Cycling relax	80.4	64.3 ± 11.5		Cycling relax	58.0	53.9 ± 8.3
	Cycling MI	74.1	59.3 ± 13.7		Cycling MI	89.7	53.0 ± 9.7
	Mean	70.7	59.1 ± 13.3		Mean	61.8	56.2 ± 9.9
U06	Static relax	84.8	60.1 ± 12.8	U13	Static relax	0.0	70.1 ± 9.2
	Static MI	57.8	56.8 ± 9.9		Static MI	100.0	59.4 ± 6.7
	Cycling relax	17.0	55.3 ± 10.6		Cycling relax	58.0	88.2 ± 7.7
	Cycling MI	88.8	56.5 ± 9.5		Cycling MI	89.7	53.0 ± 10.3
	Mean	62.1	57.2 ± 8.6		Mean	61.9	67.7 ± 12.6
U07	Static relax	99.1	63.8 ± 12.4	Average	Static relax	67.1	59.7 ± 10.5
	Static MI	76.7	70.4 ± 12.4		Static MI	65.1	57.2 ± 11.3
	Cycling relax	67.9	70.0 ± 6.8		Cycling relax	58.2	64.1 ± 11.4
	Cycling MI	82.8	69.0 ± 5.6		Cycling MI	66.3	55.2 ± 12.1
	Mean	81.6	68.3 ± 11.4		Total mean	64.2	59.1 ± 11.3

The data delineated in the table reflects the average classification accuracy derived from all individual trials for each user for both motion conditions, static and cycling; as well as the standard deviation (STD) of the accuracy among trials with CSP-LDA cross-fold validation method.

### 3.2 Frequency bands ablation study

Regarding the assessment of the impacts of various frequency bands of interest, we conducted an ablation study involving three users, as detailed in Section 2. The results of this concise study are succinctly presented in Table 3, distinguishing again four postulations (static relax and MI and cycling relax and MI). Apparently, the choice of frequency bands for the preprocessing chain does not yield significant differences in the analysis. However, it is noteworthy that the mid-range band (25Hz-50Hz) appears to play a pivotal role in motor imagery decoding, as the accuracy experiences a notable decline upon the removal of this frequency band. It is also remarkable the increase of the instability in the classification results for the static models.

**Table 3:** Results of the frequency bands ablation study.

**Frequency bands [Hz]**	**Task (% accuracy)**	**U01**	**U07**	**U10**	**Mean**
02-14—14-25	Static relax	100	96.4	75.0	90.5
	Static MI	0.9	81.0	25.0	35.6
	Cycling relax	40.2	58.9	39.3	46.1
	Cycling MI	86.2	76.7	47.4	70.1
	Mean	56.6	78.3	46.5	60.5
02-14—14-25—50-75	Static relax	97.3	87.5	62.5	82.4
	Static MI	21.6	67.2	71.6	53.5
	Cycling relax	58.0	57.1	60.7	58.6
	Cycling MI	75.9	77.6	48.3	67.2
	Mean	62.9	72.4	60.6	65.4
02-25—25-50	Static relax	69.6	96.4	71.4	79.2
	Static MI	35.3	69.0	62.1	55.5
	Cycling relax	57.1	61.6	44.6	54.5
	Cycling MI	85.3	84.5	43.1	71.0
	Mean	61.8	77.9	55.3	65.0
25-50—50-75	Static relax	0.0	91.1	58.0	49.7
	Static MI	100.0	83.6	34.5	72.7
	Cycling relax	87.5	78.6	53.6	73.2
	Cycling MI	87.9	82.5	44.7	71.6
	Mean	69.3	84.9	45.4	66.5

The values represent the classification accuracy achieved using the IFNet classifier for each preprocessing scenario and for every user in the study for both motion conditions, static and cycling, as well as the average accuracy results.

## 4 Discussion

### 4.1 Classifiers

As elaborated in Section 3.1, Deep Learning classifiers demonstrate moderately superior accuracy in the decoding of motor imagery from EEG signals compared to feature extractors-based methods. Specifically, the IFNet classifier enhances results by 5% when compared to the CSP-LDA method, as illustrated in Table 2 and Figure 8. This phenomena is repeated in three out of the four scenarios, as only the cycling relax data generates higher average accuracy outcomes with CSP-LDA classifier. However, a notable challenge arises in the form of a substantial imbalance between the decoding classes when using the IFNet classifier, rendering it less suitable for real-time classification due to its inherent instability. Users like U01 or U13, exhibiting 100% accuracy for static motor imagery but 0% for static relax, or U11 displaying 99.1% for cycling relax despite only achieving a 3.5% average accuracy for cycling motor imagery, involve totally or partially polarized decoding models with limited usability. And this issue is observed across various users, to varying degrees. We conducted several tests involving the augmentation of kernel size prior to neural network training, which mitigated this bias in results, albeit only for a limited number of users, especially when no previous scaling was applied to the data. These observations suggest the existence of additional complexities or obstacles that hinder neural networks from consistently converging when tasked with decoding motor imagery from EEG signals.

Upon analyzing Figure 8, a conspicuous correlation emerges between the two classifiers, IFNet and CSP-LDA, in terms of the accuracy observed for each user. Notably, Figure 8 shows how high-performing users achieve superior results with both classifiers, while those with average performance exhibit similar outcomes in both methods. This correlation can also be extended to the cycling models, as depicted in Figure 8, even though the static models do not manifest this trend as distinctly (Figure 8). This observation implies that users with higher performance levels may exhibit distinct EEG signal patterns or idiosyncrasies that contribute to the enhanced classifier performance, especially during motion. In this context, DL methods offer a significant advantage when working with large datasets as they can leverage the entire dataset for training models using Transfer Learning techniques. Consequently, incorporating data from exceptional users during the training of models for users with average performance can help mitigate issues related to task classification imbalance. Nevertheless, in situations where collecting extensive datasets is challenging, the significantly shorter training times offered by conventional signal processing methods like CSP-LDA and the higher stability rates they exhibit make these strategies a viable option to consider, even though they may yield slightly lower accuracy.

A brief statistical analysis has been conducted on the accuracy results, employing the Wilcoxon test to compare both classifiers in the context of both mental tasks, relaxation, and motor imagery, as well as both conditions, static and cycling. The *p*-values obtained have been adjusted using the Benjamini-Hochberg correction, resulting in a corrected p-value of 0.68 for the relax task and 0.17 for the motor imagery task, along with 0.17 for static classifiers and 0.34 for cycling ones. These indices suggest no statistical evidence to reject the null hypothesis, indicating no significant performance differences between CSP-LDA and IFNet classifiers under any task or performing condition. This result further supports the conclusion of favoring CSP-LDA for small datasets due to its shorter computing times and greater stability, with no significant functional distinctions from the IFNet classifier. However, IFNet should be considered a preferable option when working with large datasets, especially with the implementation of an inter-subject fine-tuning algorithm.

In relation to Table 1 and the MI indexes obtained by the users, it appears that there is limited correlation with their MI performance. Even so, considering the application of inter-user modeling techniques, it becomes crucial to provide clear and consistent instructions for the motor imagery task before conducting experiments. This standardization will not only enhance the accuracy achieved by Transfer Learning models but also facilitate real patients in their therapy, promoting a healthier and more effective rehabilitation process.

For future works or analyses, it is imperative to address the instability observed in the decoding models. Techniques such as fine-tuning, particularly in an inter-subject context, are posited as potentially effective means to tackle this issue; although exploring alternative decoding techniques may also offer solutions to this problem. Given the relatively recent emergence of neural networks in this field, there remains substantial room for improvement, unlike more “traditional” techniques such as CSP-LDA, deeply studied. Pioneering general EEG analysis neural networks like EEGNet (Lawhern et al., 2018) or DeepConvNet (Schirrmeister et al., 2017), among others, have progressively evolved into more specialized networks tailored for concise analysis. IFNet (Wang et al., 2023) is specifically designed for MI decoding through EEG signals, as its predecessor MIN2Net (Autthasan et al., 2021), but there also exist another approaches for EEG analysis based in Deep Learning, i.e., Bayesian-optimized Interpretable Convolutional Neural Network (BO) ICNN for P-300 analysis (Borra et al., 2022) or the CNN proposed for motor execution decoding from EEG by Borra et al. (2019). Learning from the strengths of these diverse approaches and highlighting and sharing their respective advantages could be pivotal for advancing research in EEG analysis. This collaborative approach may contribute to the ongoing refinement of decoding models and foster innovation in the field of EEG signal processing.

In this context, gaining insights into the current landscape of pedaling motor imagery decoding through EEG signals is crucial to assessing the quality of results and understanding the significance of the findings in this study. The overall average accuracy values obtained, with IFNet at over 65% and CSP-LDA at 60%, align reasonably with outputs from similar studies (Rodríguez-Ugarte et al., 2017; Ortiz et al., 2019b; Romero-Laiseca et al., 2020). However, the instability present in part of the IFNet models impacts results reliability; also, other comparable studies have achieved potentially better outcomes, for example Delisle-Rodríguez et al. attained higher average accuracies in the classification for pedaling MI throughout EEG signals, despite facing similar instability challenges in some models (Delisle-Rodriguez et al., 2019). In spite of these comparisons, the unique combination of a DL approach and a feature extraction method, coupled with the incorporation of both static and cycling motion conditions as well as the ablation study, bring forth novel insights and conclusions from this study. It is important to acknowledge that each study operates within its specific conditions, making direct comparisons challenging and not always justified.

### 4.2 Frequency bands ablation study

The primary objective of the frequency bands ablation study was to ascertain the significance of different frequency bands in the decoding analysis. The ablation study reveals that, within EEG frequency ranges, there are no substantial disparities among frequency bands when decoding the relax class using the IFNet classifier. Nevertheless, it is evident that the high beta and the low gamma bands (25–50Hz) play a crucial role in the classification of MI class, as its accuracy drops by 10% when excluded from the analysis, and even by 20% if excluded high gamma too (50–75Hz). This observation aligns with existing literature, which commonly associates these spectrum bands with motor imagery tasks (Ahn and Jun, 2015; Ortiz et al., 2019a). Anyhow, the inherent instability observed in U01 leads to the creation of extremely polarized models, potentially impacting the overall average results. The accuracy of the relax task, on the other hand, remains notably consistent regardless of the specific frequency band used in the analysis. It is indeed surprising that the most favorable relax classification results are achieved when utilizing solely the low-frequency band (2–25Hz). This observation implies that this band carries significant weight in relax classification, although it is not particularly effective for decoding motor imagery tasks, aligning also with the existing literature.

Regarding the two motion conditions (static and pedaling), no significant differences seem to emerge in the results for the static one, beyond the noticeable increase in the disparity between relax and MI when excluding the mid and high frequency bands (25–50 Hz and 50–75 Hz) from the filtering process, as the results obtained in the study remain quite consistent along the four preprocessing scenarios proposed in the ablation study. Cycling models, notwithstanding, present an improvement of approximately 10% when avoiding the lowest bands (02–25 Hz) along with remarkable stability for that case, while the rest of filtering conditions yield similar lower results. These findings suggest a potential correlation between signal noise induced by cap movement during motion conditions and the high beta and gamma bands (25–50 Hz and 50–75Hz), which appear to be crucial for cycling models.

Concerning the specific criteria used to select users for this concise study, certain conclusions can be drawn from the findings presented in Table 3. Notably, the combination of the “relax band” (2-25Hz) and the “MI ban” (25–50Hz) has a profound impact in reducing instability between classes in static models. This effect is particularly conspicuous in the data from user U01, where it not only balances the classes but also enhances the overall accuracy. Conversely, the removal of the lower band (2Hz-25Hz) seems to exacerbate the bias between classes classification, although it does enhance the accuracy for the already balanced user, U07. In summary, the evidence suggests that an effective strategy is to omit the high gamma band (50–75Hz) in static models to alleviate class imbalances, even if it comes at the cost of some reduction in overall accuracy. For cycling models, the application of high gamma filtering appears to contribute to the reduction of decoding model polarization, possibly by mitigating signal noise induced by cap movement. Moreover, the elimination of the lowest band from the signal preprocessing filtering enhances average accuracy, suggesting that applying high beta and gamma bands (25-50Hz and 50-75Hz) filtering process is the most effective option for cycling condition. In any case, it's important to note that the results obtained from this brief study are preliminary, and their consistency may vary when applying inter-user techniques.

**Figure 9:**
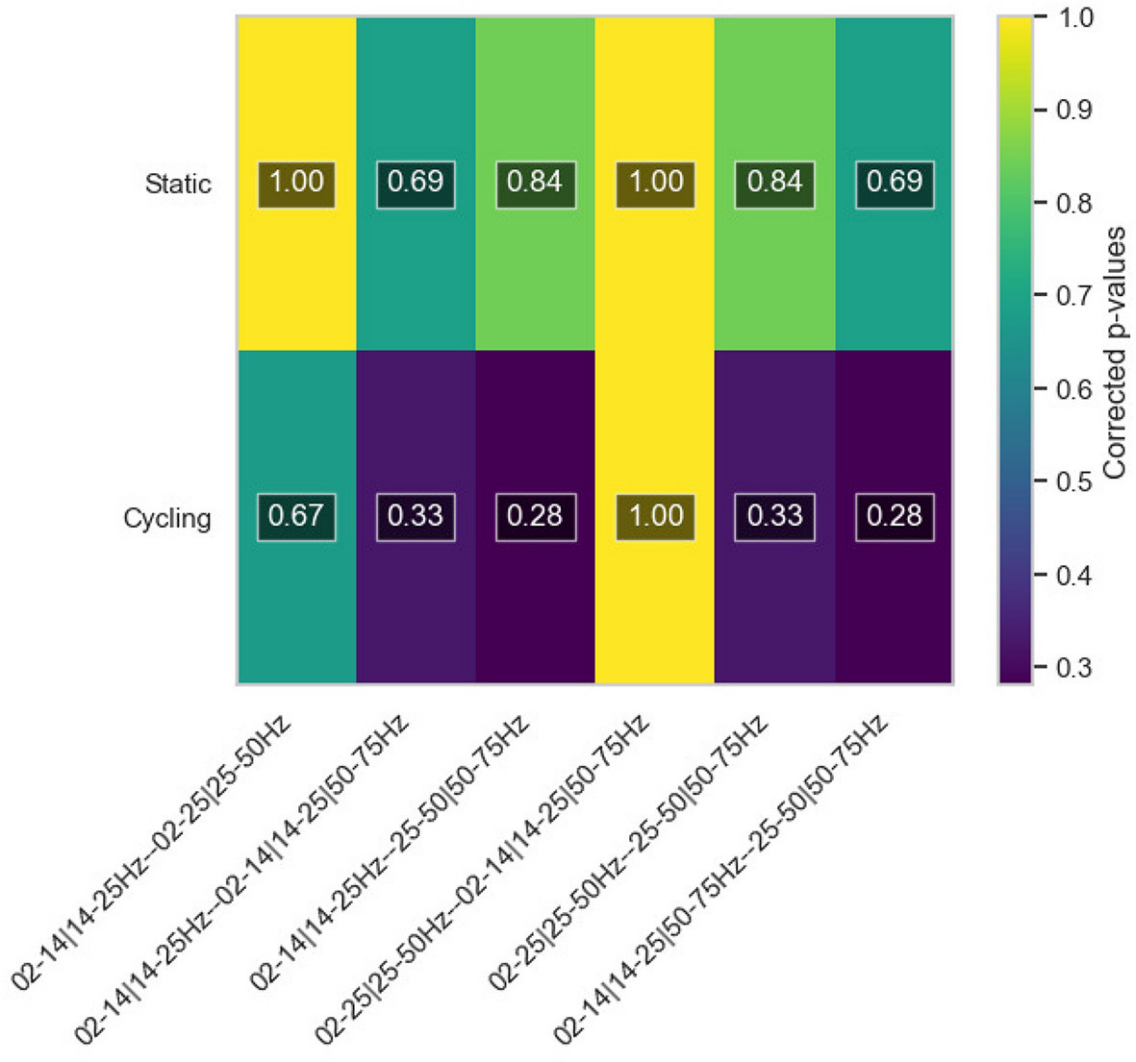
Color map of the corrected *p*-values calculated for the ablation study outputs. The map shows a comparison between pre-processing strategies for both conditions, static and cycling.

As a matter of fact, a short statistical analysis has been performed on the ablation study results, replicating the methodology outlined in Section 4.1. Corrected p-values were computed using the Wilcoxon test to compare each possible pair of the four frequency configurations utilized in the ablation analysis, this time distinguishing only between the two motion conditions, static and relax. This involved collecting data from all three participants for both relax and motor imagery tasks. The resulting p-values were then corrected using the Benjamini-Hochberg method. The outcomes of this statistical investigation are depicted in a color map, presented in Figure 9. Once again, the statistical evidence does not robustly support the conclusions drawn in the study, although they put forward stronger confidence values for cycling models, but insufficient. The comparison of 02–14—14–25—25–75Hz with 25–50—50–75Hz scenarios also yields better statistical evidence, but, again, insufficient. Nonetheless, as mentioned before, given the preliminary nature of this research involving only three users, further studies are warranted for more definitive conclusions. Notwithstanding, the findings are consistent with existing literature, suggesting that excluding the 50Hz-75Hz high gamma band to enhance stability might be a viable option for static models, as well as applying high beta and gamma bands (25–50Hz and 50–75Hz) in the filtering process for cycling conditions.

## Data availability statement

The datasets presented in this article are not readily available because the datasets generated in this study are not publicly available. Requests to access the datasets should be directed to JJ, javier.juanp@umh.es.

## Ethics statement

The studies involving humans were approved by Comité de Ética de la Investigación con medicamentos of the Hospital Universitario Severo Ochoa from Leganés (Comunidad de Madrid, Spain) under the code HLM-CYCLING-EEG. The studies were conducted in accordance with the local legislation and institutional requirements. The participants provided their written informed consent to participate in this study. Written informed consent was obtained from the individual(s) for the publication of any potentially identifiable images or data included in this article.

## Author contributions

JJ: Conceptualization, Data curation, Formal analysis, Investigation, Methodology, Software, Validation, Visualization, Writing—original draft. RM: Conceptualization, Data curation, Formal analysis, Methodology, Software, Validation, Visualization, Writing—original draft. EI: Conceptualization, Funding acquisition, Methodology, Project administration, Supervision, Validation, Writing—review & editing. MO: Conceptualization, Funding acquisition, Methodology, Project administration, Supervision, Validation, Writing—review & editing. JT: Conceptualization, Funding acquisition, Project administration, Resources, Supervision, Writing—review & editing. JA: Conceptualization, Funding acquisition, Methodology, Project administration, Supervision, Writing—review & editing.
